# Constraining the timing of the Great Oxidation Event within the Rubisco phylogenetic tree

**DOI:** 10.1111/gbi.12243

**Published:** 2017-07-03

**Authors:** B. Kacar, V. Hanson‐Smith, Z. R. Adam, N. Boekelheide

**Affiliations:** ^1^ Department of Organismic and Evolutionary Biology Harvard University Cambridge MA USA; ^2^ Department of Microbiology and Immunology University of California San Francisco San Francisco CA USA; ^3^ Department of Earth and Planetary Sciences Harvard University Cambridge MA USA; ^4^ Department of Chemistry Colby College Waterville ME USA

## Abstract

Ribulose 1,5‐bisphosphate (RuBP) carboxylase/oxygenase (RuBisCO, or Rubisco) catalyzes a key reaction by which inorganic carbon is converted into organic carbon in the metabolism of many aerobic and anaerobic organisms. Across the broader Rubisco protein family, homologs exhibit diverse biochemical characteristics and metabolic functions, but the evolutionary origins of this diversity are unclear. Evidence of the timing of Rubisco family emergence and diversification of its different forms has been obscured by a meager paleontological record of early Earth biota, their subcellular physiology and metabolic components. Here, we use computational models to reconstruct a Rubisco family phylogenetic tree, ancestral amino acid sequences at branching points on the tree, and protein structures for several key ancestors. Analysis of historic substitutions with respect to their structural locations shows that there were distinct periods of amino acid substitution enrichment above background levels near and within its oxygen‐sensitive active site and subunit interfaces over the divergence between Form III (associated with anoxia) and Form I (associated with oxia) groups in its evolutionary history. One possible interpretation is that these periods of substitutional enrichment are coincident with oxidative stress exerted by the rise of oxygenic photosynthesis in the Precambrian era. Our interpretation implies that the periods of Rubisco substitutional enrichment inferred near the transition from anaerobic Form III to aerobic Form I ancestral sequences predate the acquisition of Rubisco by fully derived cyanobacterial (i.e., dual photosystem‐bearing, oxygen‐evolving) clades. The partitioning of extant lineages at high clade levels within our Rubisco phylogeny indicates that horizontal transfer of Rubisco is a relatively infrequent event. Therefore, it is possible that the mutational enrichment periods between the Form III and Form I common ancestral sequences correspond to the adaptation of key oxygen‐sensitive components of Rubisco prior to, or coincident with, the Great Oxidation Event.

## 1 INTRODUCTION

The Precambrian evolution of the oxygenated atmosphere was strongly coupled to the evolution of oxygenic photosynthesis (Farquhar, Zerkle, & Bekker, 2011). There are numerous pathways for the uptake of carbon from the environment (Boyle and Morgan, 2011), but the Rubisco protein catalyzes the addition of CO_2_ and H_2_O to 1,5‐ribulose bisphosphate (RuBP) in the first major step of carbon fixation through photosynthesis. Rubisco also catalyzes a competing photorespiration reaction in which RuBP is combined with oxygen, which in turn reduces the overall metabolic efficiency of carbon fixation. This chemical competition is thought to derive from the relatively featureless structural attributes of CO_2_ and O_2_, which force substrate specificity to be determined largely in the transition state catalyzed by the enzyme rather than at the initial point of substrate binding (Tcherkez, Farquhar, & Andrews, 2006).

The geologic record has sufficient resolution to affirm that highly derived organisms harboring Rubisco, most notably algal and plant clades that emerged toward the end of the Proterozoic eon (Butterfield, Knoll, & Swett, 1990; Raven, Giordano, Beardall, & Maberly, 2012), have greatly impacted carbon and oxygen reservoirs and played important roles in facilitating the conversion of CO_2_ to reduced organic carbon over Earth's recent history (Mccourt, Delwiche, & Karol, 2004; Tabita, 1999). This is also presumed to be the case for Earth's more distant past with respect to photosynthetic bacteria, though perhaps to a lesser magnitude (Blank & Sanchez‐Baracaldo, 2010). However, the ability to resolve details about this distant past (specifically, when Rubisco‐mediated carbon uptake evolved or how efficiently ancestral Rubisco proteins functioned under ancient environmental conditions) are limited by the scant traces of geological and paleobiological evidence that survive from that history (Benton, Wills, & Hitchin, 2000; Braakman & Smith, 2012; Knoll, Javaux, Hewitt, & Cohen, 2006).

Rubisco genes are highly conserved and horizontal gene transfer events involving these genes are relatively rare (Tabita et al., 2007; Tomitani, Knoll, Cavanaugh, & Ohno, 2006). Age calibration of the Rubisco phylogeny against sparse geochemical and fossil records is the subject of ongoing research, and attempts have been made to map Rubisco evolution onto the substantial increase in atmospheric oxygen that occurred about 2.5 billion years ago known as the Great Oxidation Event (GOE) (Shih et al., 2016). However, the definitive cyanobacterial record only extends back to about 2.0 billion years ago (Tomescu, Honegger, & Rothwell, 2008) and it is possible that water‐oxidizing photosynthesizers existed hundreds of millions of years before the GOE (Buick, 2008; Canfield, Rosing, & Bjerrum, 2006; Crowe et al., 2013; Mukhopadhyay et al., 2014; Rosing, Bird, Sleep, Glassley, & Albarede, 2006; Stueken, Buick, Guy, & Koehler, 2015). With respect to Rubisco evolution, it is unclear exactly which genetic changes preceded or were contemporaneous with global redox changes associated with the GOE. It is also unclear if transient or localized oxygen production that preceded the GOE by hundreds of millions of years (Lyons, Reinhard, & Planavsky, 2014) could have left indelible selective marks on oxygen‐sensitive portions of Rubisco enzymes. Only by reconstructing the fullest functional range of Rubisco ancestral variation is it possible to test hypotheses or to constrain geochemical events coincident with evolutionary steps at the organismal or protein levels.

**Figure 1:**
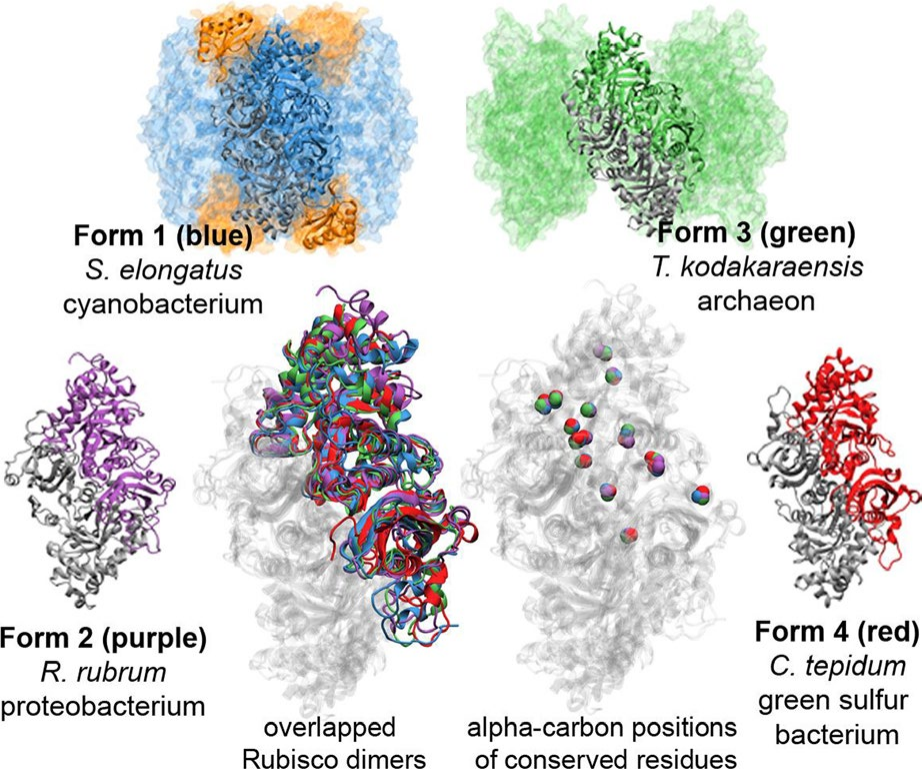
Overview of Rubisco large‐subunit dimers and dimer arrays. Representative subunits from each form (1 through 4) are color‐coded and displayed as overlapping portions of a dimer (center, left) and positions of residues with absolute conservation (center, right)

Despite the limitations of the geologic record, the extant diversity of Rubisco proteins provides a means of reconstructing elements of its role in carbon fixation by exploring its phylogeny. There are four major groups or forms of Rubisco and Rubisco‐like proteins (Figure 1). Form I is the dominant form today, as a cyanobacterial ancestor harboring a Form I Rubisco was the photosynthetic endosymbiont that eventually became the plastid of modern plants and algae (Badger & Price, 2003). Form I is a complex of eight large‐subunit dimers and eight small subunits and occurs in oxygenated environments. Form II is composed of individual dimers (comparable to the large Form I subunits) and is also found in organisms living in oxic environments such as the Proteobacteria and eukaryotic Alveolates (Tabita, Satagopan, Hanson, Kreel, & Scott, 2008). Form III is found mainly in anaerobic archaea (e.g., methanogenic and thermophilic crenarchaeota and some euryarchaeota) as either individual dimers or dimers arranged in a pentagonal array (Kitano et al., 2001). These proteins carry out the carboxylase function although the organisms that utilize Form III Rubisco fix carbon through an alternative to the Calvin‐Benson‐Bassham (CBB) pathway utilized by most photosynthetic organisms (including Cyanobacteria) to convert carbon dioxide to sugars (Aono, Sato, Imanaka, & Atomi, 2015; Sato, Atomi, & Imanaka, 2007). Form IV is a recently discovered, diverse group of enzymes referred to as Rubisco‐like proteins (RLPs). These enzymes are found within many diverse clades of organisms (including the Proteobacteria, Firmicutes, Chlorobia, Clostridia, Chloroflexi non‐methanogenic euryarchaeota, and the unicellular green alga *O. tauri*), lack the active‐site residues of canonically characterized Rubisco, and are not known to carry out the carboxylase/oxygenase activity; the full range of metabolic functions of the RLPs have not been explored but at least some are involved in sulfur metabolism (Singh & Tabita, 2010; Tabita et al., 2007).

**Figure 2:**
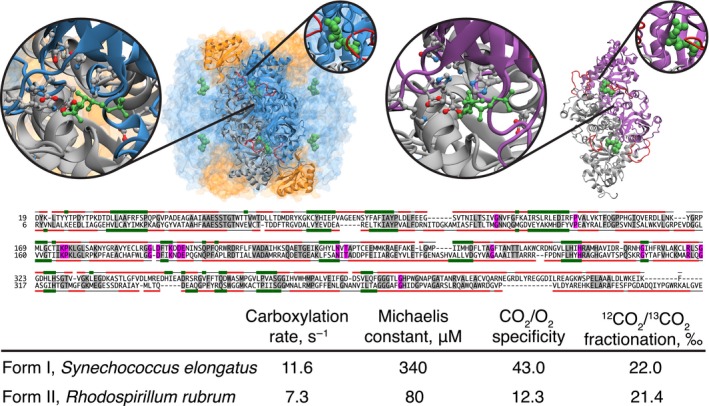
Comparison of active‐site structures and biochemical parameters of present‐day Rubisco proteins. (Top, left) Structural model of Form I from *Spinacia oleracea* (1RBL) in blue and gold. The protein is modeled in its native octomeric structure. The binding site around ribulose 1,5‐bisphosphate (RuBP) is shown in detail. RuBP is modeled in green, and ten key amino acids in the binding site are labeled. (Top, right) Structural model of Form II from *Rhodospirillum rubrum* (9RUB), with binding site detail, in purple and gray. The protein is modeled in its native dimer structure. (Center) Sequence alignment of forms I and II. Red and green bars above/below sequences indicate portions that are helices and sheets, respectively. (Bottom) Comparison of biochemical properties of forms I and II, with data from Savir et al. (2010)

Present‐day forms of Rubisco exhibit a negative correlation between maximum CO_2_ turnover rate and CO_2_/O_2_ substrate specificity; mutational variants of Rubisco can increase performance in one of these parameters at the expense of decreased performance of the other (Savir, Noor, Milo, & Tlusty, 2010). This correlation inherently restricts the overall efficiency of the enzyme and the larger metabolic system of its host organisms (Figure 2) (Portis & Parry, 2007). In response to this trade‐off between Rubisco's biochemical parameters, obligate and facultative phototrophs that have evolved varied and sophisticated active inorganic carbon transport systems, including specialized localization for both internal and external carbonic anhydrase, and localization of Rubisco within the chloroplast or cyanobacterial cell in regions where CO_2_ can be elevated (Badger et al., 1998). The relatively limited trade‐off between these kinetic properties (i.e., turnover rate and CO_2_/O_2_ specificity) of Rubisco suggests that the biochemical optimization of Rubisco function may be very ancient (Gutteridge & Pierce, 2006; Tcherkez et al., 2006).

As the bottleneck for carbon fixation in the predominant oxygenic photosynthetic pathways, Rubisco is at the heart of many fundamental questions about the co‐evolution of early life and the development of biogeochemical cycles of the planet (Gimpel, Specht, Georgianna, & Mayfield, 2013). Cyanobacteria, diatoms, plants, and algae utilize Form I Rubisco, linking the evolution of the protein to the appearance of Earth's dominant photoautotrophic organisms. By inference, Form I Rubisco has been one of the main intermediaries between carbon in the air and carbon in organic matter for much of Earth's history (Nisbet et al., 2007). Its evolution followed a prior anoxygenic history that may have been rooted in chemolithoautotrophs bearing Rubisco‐like proteins or other CO_2_‐fixing micro‐organisms that likely predated those utilizing the Calvin cycle (Ashida, Danchin, & Yokota, 2005). This interpretation is consistent with models of a linear bacterial phylogeny in which Gram‐positive bacteria emerged earlier than Archaea, Gram‐negative bacteria or photosynthetic bacteria; organisms such as *B. subtilis* that use the methionine salvage pathway with Rubisco‐like proteins would have emerged before the evolutionary completion of the Calvin cycle and thus Rubisco's canonical role in CO_2_ uptake (Gupta, 1998). Another interpretation of the available data from Rubisco and Rubisco‐like proteins posits that the most likely scenario was that a Form III Rubisco, arising within the Methanomicrobia, was the ultimate source of all Rubisco and RLP lineages (Tabita, 1999; Tabita, Hanson, Satagopan, Witte, & Kreel, 2008; Tabita, Satagopan, et al., 2008). Regardless of which part of the Rubisco protein family network emerged first, the ancestral path of Form I is the most conducive to calibration with the Precambrian fossil record over a span of time that includes the emergence of significant amounts of oxygen in the Earth's atmosphere (Schopf, 2011).

In addition to the fossil record of Rubisco‐harboring organisms, Form I enzymes also produce a characteristic carbon isotope fractionation value of about −25 parts per thousand, which is imprinted upon the fixed organic matter generated through the CBB pathway relative to background isotope fractionation values associated with carbonate rocks. The isotope discrimination signal generated by Rubisco is presumed not to have greatly changed over time and has therefore been used to interpret the consistent difference between organic and carbonate isotope values as contextual evidence for biological activity (Planavsky, Asael, et al., 2014; Schidlowski, 1988). However, there is growing evidence that the isotope discrimination values of a broader sampling of Rubisco proteins can range markedly beyond that observed for model Form I proteins. Form I homologs from the coccolithophore *Emiliana huxleyi* range down to −11 parts per thousand (Boller, Thomas, Cavanaugh, & Scott, 2011). Perhaps more pointedly, a single site substitution on a part of the Rubisco enzyme that closes around the RuBP substrate can decrease isotope fractionation for Form I Rubisco from the tobacco plant from −27 to −11 parts per thousand, and there is also evidence that carbon isotope fractionation is tightly coupled to CO_2_ specificity (Mcnevin et al., 2007). Rubisco functionality on a young Earth with abundant CO_2_ may not have experienced selective pressures for high specificity, which casts the uniformitarian assumption for Rubisco's distinct isotopic discrimination pattern into question.

Reconstructing ancestral states of Rubisco proteins through phylogenetics and the subsequent structural and biochemical characterization may provide a means of investigating the limits of the uniformitarian assumption for Rubisco's ancestral phenotype. Ancestral sequence reconstruction may help overcome uncertainties of historical information obtained primarily from fossil inferences (Benner, Sassi, & Gaucher, 2007; Felsenstein, 1981; Kacar, 2016; Kumar & Hedges, 1998; Parfrey, Lahr, Knoll, & Katz, 2011; Pauling & Zuckerkandl, 1963). This approach utilizes phylogenetic models of sequence evolution to computationally reconstruct ancestral gene and protein sequences. Reconstructed ancestral sequences can then be resurrected through in vivo or in vitro synthesis and their properties can be characterized in the laboratory (Dean & Thornton, 2007; Jermann, Opitz, Stackhouse, & Benner, 1995; Kacar & Gaucher, 2012; Kacar, Garmendia, Tuncbag, Andersson, & Hughes, 2011) Reconstruction methods may be extended to test hypotheses related to the deep evolutionary past and to identify historically significant mutation sites for genes and proteins, providing insights into the mutational basis of evolutionary innovations and sequence and structural level protein evolution through billions of years of evolutionary time (Chang, Jonsson, Kazmi, Donoghue, & Sakmar, 2002; Harms & Thornton, 2013; Kacar & Gaucher, 2013; Kacar, Ge, Sanyal, & Gaucher, 2017; Perez‐Jimenez et al., 2011; Trudeau, Kaltenbach, & Tawfik, 2016; Voordeckers et al., 2012).

In this study, we have used ancestral sequence reconstruction to infer the ancestral sequences of major nodes of the Rubisco family of proteins, extending backwards along the Form I ancestral line (which includes plants, algae, and cyanobacteria) to the confluence of the major Rubisco form groups. We have used homology modeling to predict the structure of the inferred ancestors. The overall objective is to compare inferred ancestral Rubisco genotypes and structures against observed modern homologs to pinpoint the phylogenetic location where biochemical attributes of the proteins associated with responses to oxidative stress are likely to have emerged, and to assess whether this is consistent with the timing of the GOE (Mann, Bradley, & Hughes, 1999). Here, we present the first complete array of inferred Rubisco ancestral sequences, with a focus on six reconstructed ancestors at important nodes extending along a transect starting in the oxic Form I group and ending at the common ancestor of anoxic Form III and Form IV groups. The tree and all Rubisco sequences can be readily accessed and downloaded through the PhyloBot web interface (Hanson‐Smith & Johnson, 2016).

## 2 METHODS

### 2.1 Phylogenetic reconstruction of ancestral rubisco protein sequences

Reconstructions were performed using PhyloBot software (phylobot.com) (Hanson‐Smith & Johnson, 2016). Orthologs of the Rubisco family were identified by BLAST search based on the amino acid sequences of groups IA and IB Rubisco in *Synechococcus elongatus* and group IV Rubisco in *Baccillus*, using the NCBI BLAST Tool (Altschul, Gish, Miller, Myers, & Lipman, 1990). Using 81 curated sequences, multiple sequence alignments were inferred using MSAProbs (Liu, Schmidt, & Maskell, 2010) and MUSCLE (Edgar, 2004) with the default settings. Both of these alignments were best‐fit by the PROTCATWAG model (Lartillot & Philippe, 2004; Le & Gascuel, 2008), with model fitness assessed using the Akaike information criterion (Abascal, Zardoya, & Posada, 2005). The 81 protein sequences used in this study are available to download from the following URL: http://www.phylobot.com/582058404/RuBisCO.noalign.fasta

Using WAG+G substitution model, we used a maximum‐likelihood (ML) algorithm (Yang, 1996) to infer the ancestral amino sequences with the highest probability of producing all the extant sequence data. Specifically, we used RAxML version 7.2.8 to infer the ML topology, branch lengths, and evolutionary rates (Stamatakis, 2006). We exported this ML phylogeny to another software package, PhyML (Guindon et al., 2010), in order to calculate statistical support for branches as approximate likelihood ratios. We next reconstructed ML ancestral states at each site for all ancestral nodes using the software package Lazarus (Hanson‐Smith, Kolaczkowski, & Thornton, 2010). We used sequences from the group IV family as the outgroup to root the tree. We placed ancestral insertion/deletion characters according to Fitch's parsimony (Fitch, 1971), in which each indel character was treated independently.

We extracted the ancestral sequences from the phylogenetic nodes corresponding to several relevant ancestors between and within the Rubisco form groupings. We named each of these five ancestors according to their descendant sequences. For example, the ancestor named Anc. I/II/III is the most recent shared ancestor of sequences from groups 1, 2, and 3. Similarly, Anc. I is the most recent shared ancestor of sequences from Group 1. The extracted ancestors include Anc. I/II/III, Anc. I/III, Anc. I/III’, Anc. I, Anc. IAB and Anc. IB. We characterized the support for these ancestors by binning their posterior probabilities of states into 10% sized bins and counting the proportion of ancestral sites in each bin. We also generated alternate versions of the ancestral sequences by randomly sampling from their posterior distributions to generate five alternate ancestors for every node, as described (Williams, Pollock, Blackburne, & Goldstein, 2006).

### 2.2 Homology modeling of ancestral rubisco proteins

Atomic‐level structural models of five ancestral Rubisco proteins—the MRCA of all group 1B sequences (Anc. IB), the MRCA of groups 1B and 1A (Anc. IAB), the MRCA of Group 1 (Anc. I), the MRCA of groups 1 and 3 (Anc. I/III), and the MRCA of groups 1, 2 and 3 (Anc. I/II/III)—were generated using homology models based on known structures of Rubisco catalytic subunits. Twenty‐three template structures were selected from the Protein Data Bank based on sequence continuity, the conformational form (employing the activated or inhibitor‐bound forms when available) and wild‐type enzymes. The PDB acquisition codes of these structures are as follows: 1BWV, 1BXN, 1GK8, 1IR1, 1RSC, 1SVD, 1TEL, 1WDD, 2D69, 2OEK, 2OEL, 2OEM, 2QYG, 3A12, 3ZXW, 4F0M, 4HHH, 4LF1, 4LF2, 4MKV, 4NAS, 4RUB, 9RUB. These template structures were prepared by removing all but one catalytic dimer, by removing all ligands and crystallographic ions and solvent, and by removing atoms to convert modified residues to their parent residues. An alignment with the template structures was generated for each MLSA using SWISS‐PDB Viewer (Guex & Peitsch, 1997) with the MLSA threaded to the superimposed templates. Spatially restrained homology models based on these alignments were generated using Modeller 9.15 (Sali & Overington, 1994) with the positions of the alpha carbons constrained to maintain backbone symmetry across the two chains of the dimer. The quality the predicted structures based on steric clashes and protein geometry was confirmed to lie within the quality of the template structures using the MolProbity web interface (Chen et al., 2010).

### 2.3 Definition of dimer structural domains

Nine regions of the catalytic dimer were defined based on their relevance to the tertiary structure and the biochemistry of the enzyme. These regions are as follows: the interface between large subunits in Form I Rubisco, the interface between large and small subunits in Form I Rubisco, the interface between large subunits in Form III Rubiscos, the interface between chains in the catalytic dimer, the strands in the α/β barrel that caps the active site, loop 6 which closes the active site in the enzyme's activated state, and the C‐ and N‐terminal domains. Residues were defined to be at an interface if any non‐hydrogen atom was within a cutoff distance (<5 Å to include nonpolar interactions) of an atom at the opposite side of the interface. For interfaces in the Form I and Form III tertiary structures, these distances were measured between residues in the ancestral dimer superimposed on the dimer in an existing enzyme (Form I: pdb code 1RBL from *Synechococcus elongatus*; and Form III: pdb code 1GEH from *Thermococcus kodakaraensis*) and residues in the respective chain of the existing enzyme. The residues in the strands in the α/β barrel, in loop 6, and in the C‐ and N‐termini were defined as those whose alpha carbon was closest to the alpha carbon of a residue within those subdomains in *S. elongatus* Rubisco after superimposing the two dimers. Residues are defined to be near the α/β barrel if they are within the cutoff distance of the strands in barrel as defined above.

### 2.4 Tests for substitution enrichment

For each structural region (see Figure 1), we tested the extent to which it was enriched for amino acid substitutions during five historic phylogenetic windows. These windows are defined as (i) the branches connecting Anc. I/II/III to Anc. I/III, (ii) the branches connecting Anc. I/III to Anc. I/III’, (iii) the branches connecting Anc. I/III’ to Anc. I, (iv) the branches connecting Anc. I to Anc. IAB, and (v) the branches connecting Anc. IAB to Anc. IB. We then applied the Fisher's exact test as follows. We first compared the maximum‐likelihood ancestral sequences at either end of each phylogenetic window and counted the number of amino acid sites in four different categories: (i) sites with an amino acid substitution and in the structural region of interest, (ii) sites without a substitution and in the structural region, (iii) sites with an amino acid substitution and not in the structural region, and (iv) sites without a substitution and not in the structural region. We then applied the Fisher's exact test, using the four count values as in the input matrix. We applied this test for every combination of structural region and phylogenetic window and collected the odds ratios and *p*‐values from the test. We defined a structural region to be significantly enriched for mutations in a phylogenetic window if its odds ratio is greater than 1.5 and its *p*‐value is less than .05.

## 3 RESULTS

### 3.1 Reconstructing ancestral rubisco protein sequences

**Figure 3:**
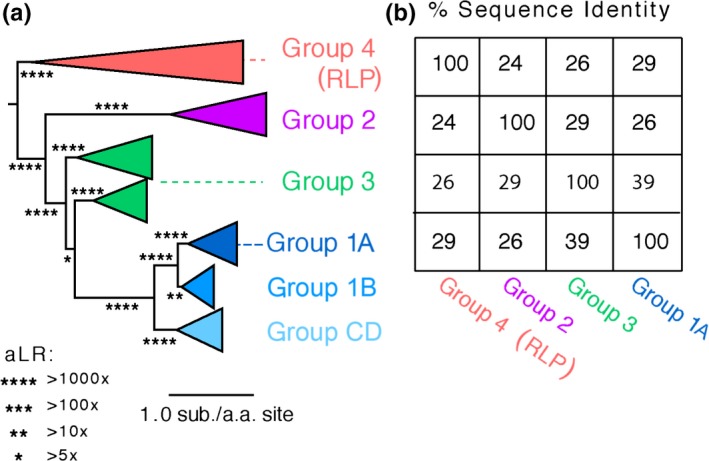
Maximum‐likelihood Phylogeny and Sequence Identity Between Rubisco Subgroups. (a) From the Rubisco protein family phylogeny, four representative extant sequences were selected: *Chlorobium tepidum* from Group 4 and Rubisco‐like proteins (RLP), *R. rubrum* from Group 2, *Thermococcus kodakaraensis* from Group 3, and *Synechococcus elongatus* from Group 1A. The branch lengths express substitutions per amino acid site. The stars on branches express their support as approximate likelihood ratios (aLR). (b) The table expresses the percent identity between all pairs of the four representative sequences

The library of ancestral Rubisco sequences was constructed using the PhyloBot software, and can be viewed at the following URL: http://www.phylobot.com/rubisco.v4/ (Hanson‐Smith & Johnson, 2016). Based on a library of eighty‐one present‐day Rubisco large subunit (rbcL) and Rubisco‐like protein sequences, we reconstructed a maximum‐likelihood (ML) phylogeny of the Rubisco protein family. The ML phylogeny supports an evolutionary history in which Rubisco forms I, II, and IV are each distinct evolutionary groups, and Form III is paraphyletic with respect to Form I as a subgroup (Figure 3a, Tables S1 to S4). Although the sequence identity of present‐day Rubisco proteins is relatively low across groups (Figure 3b), the maximum‐likelihood reconstruction provides relatively strong support for the separation of the groups into clades. The sequences within the group I clade, which includes cyanobacteria, some bacteria and most photosynthetic eukaryotes such as the green plants, green algae, red algae, euglenozoa, and stramenopiles, are strongly supported to be monophyletic (aLR = 1.05 × 10^83), with relatively strong support for the monophyly of subgroups IA, IB, and IC/D. The group II sequences, including many proteobacteria and some eukaryotic alveolates, are also strongly supported to be monophyletic (aLR = 4.36 × 10^71). The best‐fitting evolutionary model split the sequences from Group 3 into two distinct clades of Archaea that include methanogens and extremophiles. Each of these clades is strongly supported (1.71 × 10^4 and 6.96 × 10^5, respectively), but the support for the paraphyletic group III split (which we refer to as Ancestor or Anc. I/III) is relatively less certain (aLR = 5.08).

**Figure 4:**
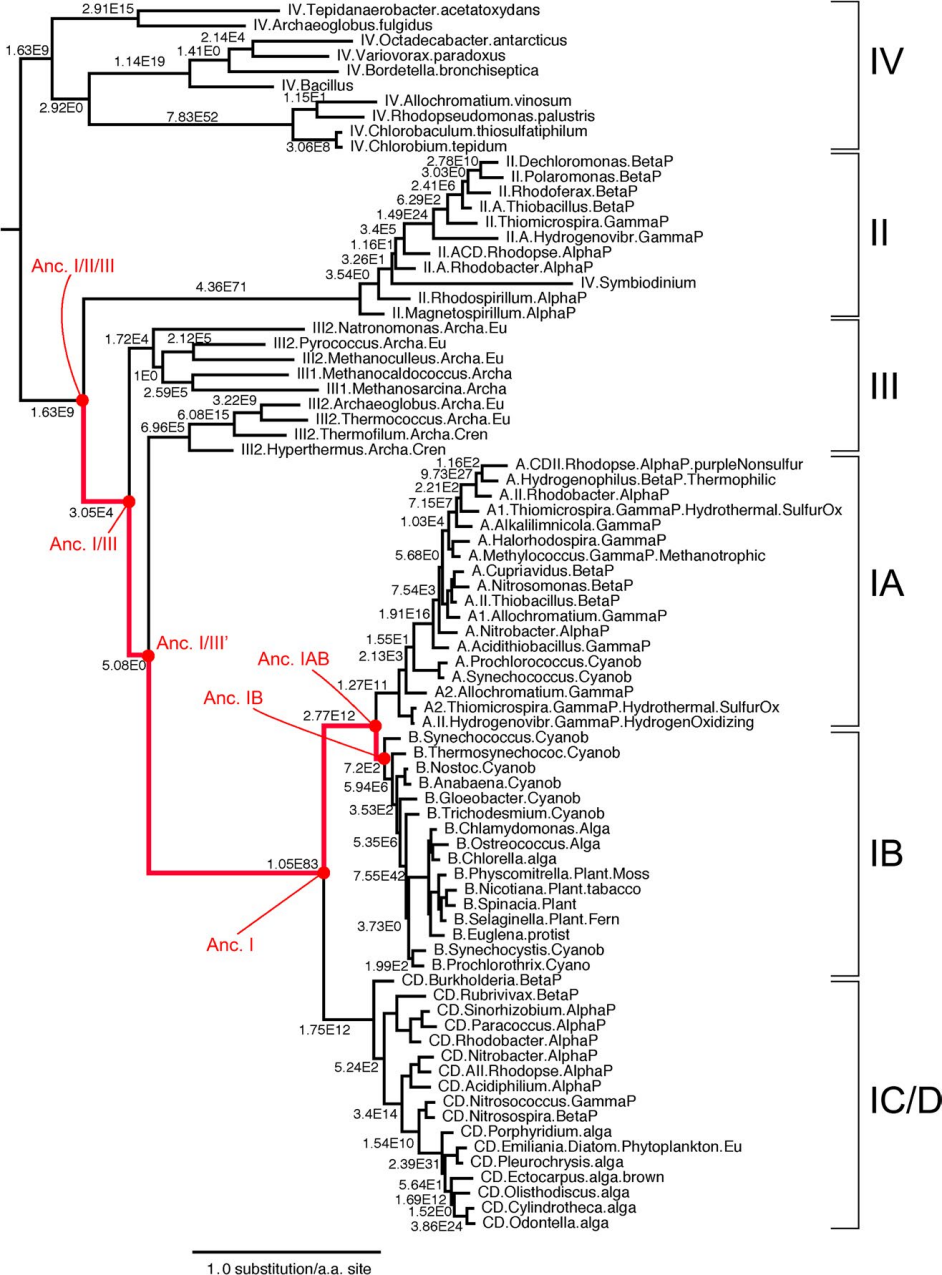
Maximum‐likelihood Phylogeny of Rubisco Protein Family. Branch lengths express amino substitutions per sequence site. Decimals on internal branches express the approximate likelihood ratio of the branch existence, compared to the next best hypothesis in which the branch does not exist. Six ancestors, on internal nodes, are labeled along an evolutionary trajectory from the most recent common ancestor (MRCA) of groups 1, 2, and 3 to the MRCA of Group 1B. Ancestral sequences are labeled according to their membership in Rubisco subfamilies. The phylogenetic positions of ancestral proteins relevant to this study are labeled in red

We next reconstructed protein sequences at all internal nodes of the ML phylogeny, using an empirical Bayesian approach that predicts the probability of all possible twenty amino acids at every site in the protein sequence (Yang, 1996). The reconstructed protein sequences correspond to ancient (extinct) proteins that were ancestral to various groups of present‐day Rubisco proteins. We identified five ancestors along an evolutionary trajectory that starts at the most recent common ancestor of groups I, II, and III and extends to the MRCA of the group I clade (Figure 4). Every ancestor is represented as a two‐dimensional matrix of amino acid probabilities *p*, where *p*(i,j) is the probability of amino acid i at site j. A maximum‐likelihood protein sequence can be extracted for every ancestor by taking the amino acid with the highest probability at every site (Table S2).

### 3.2 Structural analysis

We built structural homology models of ancient Rubisco proteins based on the reconstructed ancestral sequences and crystallographic atomic structures of present‐day Rubisco proteins. We colored regions of these structural models based on their structural similarity to present‐day Rubiscos (Fig. S1). This coloring reveals that tertiary aspects of all present‐day Rubisco proteins should be found, to varying extents, in reconstructed Rubisco structures.

### 3.3 The evolution of interaction domains

**Figure 5:**
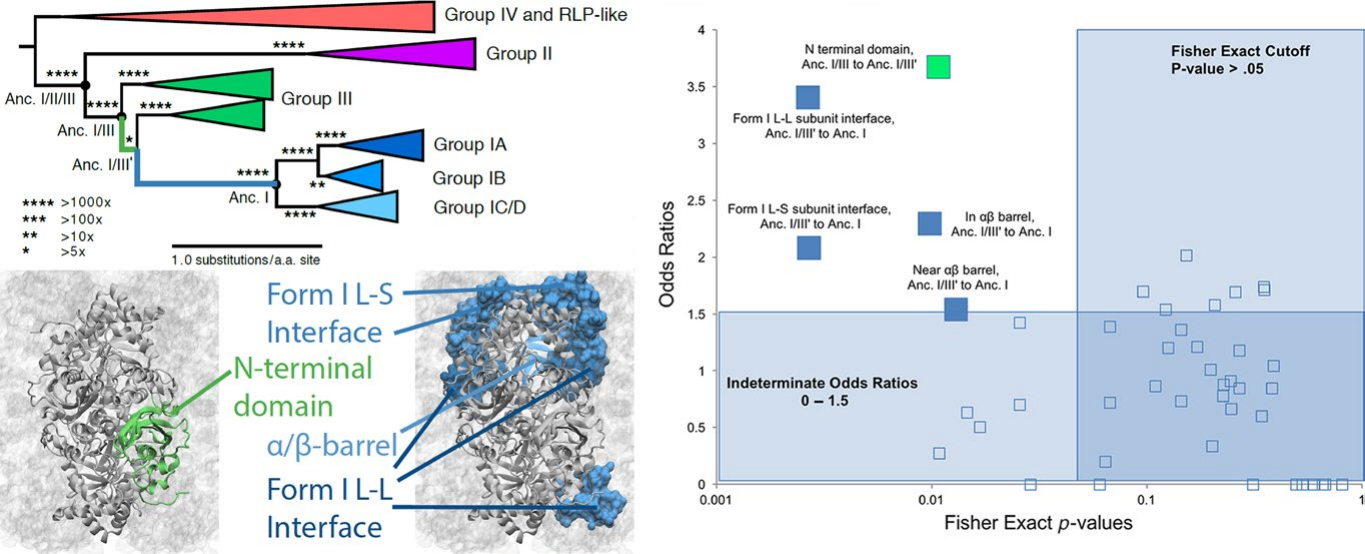
Top, left: Pictographic representation of the Rubisco phylogenetic tree. Branch lengths indicate average amino acid substitutions per sequence site, and stars indicate the strength of phylogenetic support. Branches are color‐coded to correspond with Rubisco structural portions (bottom, left) that exhibit statistically significant mutation rates. Right: scatter plot of all Rubisco ancestors and structures analyzed using the Fisher's exact test. Ancestors and structures with significant mutational enrichment color‐coded as in the tree figure at top left

A comparison of ancestral protein sequences and structures along phylogenetic branches reveals that some branches are enriched for amino acid substitutions in specific structural regions, including the small‐subunit interfaces, large‐subunit interfaces, dimer interfaces, and activation site (Tables S1 to S4). We defined several functionally relevant structural regions within the Form I and Form III Rubisco structures (Figure 5). We then counted the number of amino acid substitutions that occurred within each of those regions on branches, and statistically tested if each region–branch combination was enriched for substitutions compared with the rest of the protein sequence (Methods). Specifically, on the branch leading to the ancestor of all Form I sequences, amino acid substitutions were 2.9 times more prevalent within the large‐subunit interface (*p* = .078), 3.8 times more prevalent within the small‐subunit interface (*p* = .001), and 2.3 times more prevalent in the AB barrel (*p* = .01). Similarly, on an ancestral branch that splits the Form III clade, there was 3.7 times enrichment for amino acid substitutions in the N‐terminal domain (*p* = .01). Taken together, these tests reveal that several functionally important protein regions experienced punctuated historic periods of increased substitution rates.

## 4 DISCUSSION

In this study, we used the methods of phylogenetic ancestral sequence reconstruction to computationally infer amino acid sequences and structures for ancient Rubisco proteins that represent the ancestors of all known Rubisco forms.

Our phylogenetic analysis supports an evolutionary interpretation in which Group III is most closely related to Group I. This interpretation is consistent with previous phylogenetic analysis in which protein sequences of Rubisco Group I and Group III were inferred to form a monophyletic clade exclusive of Group II and IV (Tabita, Satagopan, et al., 2008). However, there exists an alternate hypothesis in which Group II—not Group III—is most closely related to Group I (Andersson & Backlund, 2008; Ashida et al., 2005). Given that the true evolutionary history of Rubisco cannot be observed, we are left to discriminate between these two hypotheses based on phylogenetic signal and a biophysical comparison of extant Rubisco protein species. Based on our maximum‐likelihood phylogenetic analysis and the similarity of the inferred tertiary structures, the evolutionary hypothesis in which groups I and III are sisters is 3.05 × 10^4 more likely than the next best hypothesis in which groups I and II are sisters. Although future phylogenetic analysis—using larger numbers of sequences or more complex evolutionary models—may revise the relative weight for these competing hypotheses, in the meantime the phylogenetic evidence seems to be overwhelmingly in support of an evolutionary scenario in which Group II branched separately from the clade containing the ancestors of groups I and III.

Previous work with ancestral Rubisco has focused on smaller subfamilies of protein sequences and structures, leading to reconstructed ancestral sequences that differ from those found in our study. Studies of Rubisco evolution focused on the emergence of CO_2_ management in recently derived C4 photosynthetic plants (Christin et al., 2008), the evolutionary pathways of thermostable Rubisco homologs (Miller, Mcguirl, & Carvey, 2013), and the underlying trade‐offs between destabilizing mutations and environmental tolerance in Rubisco functionality (Studer, Christin, Williams, & Orengo, 2014). Recently, Shih et al. (2016) reconstructed recently derived Rubisco in order to probe Precambrian adaptations to increased O_2_ and decreased CO_2_ surrounding the GOE, but their approach to phylogenetic reconstruction differed from ours in several critical ways. First, we sampled a much broader range of Rubisco forms (including sequences from the anoxic forms III and IV), which allows for greater confidence in rooting the phylogeny and inferring directionality of amino acid substitutions. Shih et al. chose as their phylogenetic outgroup Form I sequences from relatively sophisticated algae (i.e., recently derived *Porphyra, Porphyridium,* and *Cyanidioschyzon*). It is unlikely that their choice of outgroup can effectively probe Rubisco's adaptive response from anoxic to progressively more oxic states, calling into question the mapping of their findings to the GOE. In contrast, we rooted our tree with present‐day sequences broadly sampled from anoxic Rubisco forms III and IV. In theory, this increases the accuracy of our “deep” ancestral reconstructions by breaking up long branches. Finally, we compared our ancestral sequences across the different phylogenetic models (see 2), which allowed us to examine the extent to which our ancestral reconstructions were robust across models rather than artifacts of any particular model.

In this study, we used the Fisher's exact test to identify structural regions and phylogenetic branches enriched for amino acid substitutions compared to the background rate of substitution. Our approach should not be confused with the dN/dS test, which seeks to identify sequence sites under positive selection (Yang, 1996). Unlike the dN/dS test, our application of the Fisher's exact test does not enable conclusions to be drawn about the strength of selection (Mugal, Wolf, & Kaj, 2014). Rather, our test simply identifies sequence regions with elevated substitution rates and allows for the possibility that these elevated rates could be due to increased selection, relaxed selection, or possibly other causes. Given that our study is primarily concerned with what caused selection to occur—rather than the mode of selection that actually occurred—the Fisher's exact test is an ideal statistical mechanism for examining relative substitution rates between different parts of the protein and different phylogenetic branches.

In light of the inherent difficulty of mapping Rubisco to a poorly calibrated geochronological record, we focused on five historic phylogenetic windows along the evolutionary trajectory leading from the Form IB ancestor to the Form I/II/III ancestor. Though the actual root sequence of Rubisco is unknown, the evolutionary trajectory leads from recent conditions that approximate the state of the ancestral Group IB enzyme just prior to the primary endosymbiosis event that gave rise to the green algae to more ancient, obligate anoxic conditions (i.e., an ancestor that predates the development of both of the oxic Form I and Form II Rubisco groups). We then binned all amino acid substitutions that occurred along this lineage by their location in various tertiary structures: the Form I large–large subunit interface, the Form I large–small subunit interface: the Form III large–large subunit interface, the dimer interface, sites near the alpha‐beta barrel, sites within the alpha‐beta barrel, loop 6 of the helix that shepherds the substrate near the active site, the N‐terminal domain, and the C‐terminal domain.

Our analysis of amino acid substitution enrichment along the selected phylogenetic transect suggests possible relationships between the geochemical history of the Earth and the mutational history of Rubisco. The branch connecting the Ancestor (Anc.) I/II/III node to the Anc. I/III node is not significantly enriched for amino acid substitutions as measured by the Fisher exact *p*‐values. However, on the branches connecting the Archaeal clade ancestors (Anc. I/III) to an intermediate Archaeal clade that preceded the emergence of Group I (Anc. I/III’), the N‐terminal domain exhibits a significantly enriched rate of amino acid mutation fixation. The N‐terminal domain of the large subunit is not directly involved in CO_2_ fixation; however, it does associate with the C‐terminus of the neighboring large subunit within the dimer and is therefore involved in the assembly process (Schneider, Lindqvist, Branden, & Lorimer, 1986; Schneider et al., 1990). It comprises an RNA recognition motif that becomes exposed under oxidizing conditions when the glutathione pool shifts toward its oxidized form (Cohen, Sapir, & Shapira, 2006). The oxygen sensitivity of the N‐terminus, and its role binding to RNA, may play an evolutionarily significant role in regulating Rubisco expression (Cohen et al., 2006; Kapralov & Filatov, 2007; Yosef et al., 2004). Regulation of expression can attenuate acute physiological response to perturbation inside or outside the cell (Bailey, 1991; Berry, Mure, & Yerramsetty, 2016). For these reasons, it is possible that this substitutional enrichment associated with the N‐terminus may reflect an initial adaptive attempt to cope with increasing oxidative stress prior to the GOE. (Cohen et al., 2006).

It is unknown exactly when the GOE occurred relative to the reconstructed Rubisco ancestors, but the relatively long branch (approximately 0.8 substitutions per site) leading to Form I and the portions of the Rubisco large subunit that underwent mutational enrichment suggest that the GOE could have occurred in the phylogenetic window between Anc. I/III and Anc. I. Indeed, the Anc. I node exhibits very high sequence similarity to sequences found in obligate aerobes such as *Burkeholderia* (formerly within *Pseudomonas*), *Tropicibacter* and *Synechococcus*, each with about 78% exact sequence identity and 1–2% residue gaps separating these extant organismal sequences from the reconstructed node. Conversely, the Anc. I/III node exhibits similar sequence identities and gaps (77–83% and ~1%, respectively) with thermophilic, autotrophic and anaerobic *Crenarcheota* such as *Hyperthermus, Pyrodictium,* and *Pyrococcus*; the Anc. I/III node sequence more closely resembles Form III sequences from anoxic organisms, despite the long branch lengths from this node to either extant Form I or Form III homologs. Our interpretation based on the sequence similarities on either side of this window is further supported by the similarities of the tertiary structures to those of modern homologs (Fig. S1). Most residues in the Anc. I/III structure are proximal to locations associated with anoxic forms III and IV (green and red colored residues, respectively), but nearly all residue locations of the Anc. I ancestor correspond with positions associated with a typical Form I structure. Within this phylogenetic window, the Form I large–large and Form I large–small subunit interfaces are significantly enriched for amino acid substitutions, in addition to weaker enrichments associated with the dimer interface and residues both near and within the alpha‐beta barrel where most of the catalytic residues are located (Chapman et al., 1988). These structural regions are not enriched for substitutions in the other four phylogenetic windows. We interpret these respective sequence similarities, and the mutational enrichments in oxygen‐sensitive catalytic structures of the large subunit along this branch, to indicate that the GOE is more likely to correlate with sequences between these two nodes, rather than near the nodes proximal to the appearance of cyanobacterial clades.

Our interpretation implies that the periods of Rubisco substitutional enrichment inferred near the transition from anaerobic to aerobic physiologies predate the acquisition of Rubisco by fully derived cyanobacterial (i.e., dual photosystem‐bearing, oxygen‐evolving) clades. The partitioning of extant lineages at high clade levels within our Rubisco phylogeny indicates that horizontal transfer of Rubisco is a relatively infrequent event. Therefore, it is possible that these mutational enrichment periods correspond to the adaptation of key oxygen‐sensitive components of Rubisco prior to the GOE (Anbar et al., 2007; Planavsky, Reinhard, et al., 2014). This would further indicate that calibrating the Rubisco tree to the appearance of cyanobacterial fossils or the GOE itself must be undertaken with care, given the possibility that stem group oxygenic photosynthetic organisms could have existed long before the appearance of recognizable Cyanobacteria in the fossil record (Blankenship & Hartman, 1998; Cardona, 2016; Fischer, Hemp, & Johnson, 2016; Johnson et al., 2013). Phenotypic characterization of expressed and purified ancestral forms of Rubisco may provide a biochemical and physiological basis for correlating the specific site mutations between the Anc. I/III and Anc. I branches with adaptations to oxidative stress.

Clearly, caution must be exercised when interpreting a complex history of interactions between geological and biological processes through the lens of a single gene or enzyme, even one as critical and well characterized as Rubisco. However, lines of evidence from extant organismal physiology and Precambrian geochemical indicators corroborate the possibility that some rise in oxygen or other oxidized chemical species preceded the emergence of Form I Rubisco enzymes within cyanobacterial clades. Previous phylogenetic analyses indicate that anoxygenic photosynthetic lineages are more deeply rooted than oxygenic cyanobacterial lineages (Mulkidjanian et al., 2006; Xiong, 2007) and that cyanobacteria represent an evolutionary intermediate between anaerobic and obligate aerobic organisms (Harel, Karkar, Cheng, Falkowski, & Bhattacharya, 2015). Co‐evolution at organismal (i.e., the emergence or development of localized CO_2_ or O_2_ control volumes within cells) and protein (i.e., direct accumulation of mutations in sequences representing oxygen‐sensitive regions of proteins) levels may have been tightly coupled just prior to the GOE due to oxygen stresses and diminishing CO_2_ availability in the near‐surface environment (Knoll, 2006; Tomitani et al., 2006). The oceans of the Archean and early Proterozoic were laden with Fe^2+^, and it has been proposed that iron‐oxidizing photosynthetic organisms comparable to Proteobacteria or Chlorobi could have dominated the photic zone, driving the widespread deposition of banded iron formations (Kappler, Pasquero, Konhauser, & Newman, 2005). Prior to the buildup of atmospheric oxygen, facultative oxygenic photosynthesizers would have competed much more directly with obligate anoxygenic photosynthesizers (Gupta, Mukhtar, & Singh, 1999), exploiting a similar range of reduced electron donors (Cohen, Jorgensen, Revsbech, & Poplawski, 1986), but with the added (though inefficient) capacity to draw on water as other sources became locally exhausted (Butterfield, 2015; Johnston, Wolfe‐Simon, Pearson, & Knoll, 2009). These interactions leave ample room for investigation regarding the timing, ecological relationships and intermediate stages in the development of fully derived, oxygenic photosynthesizers that may be reflected in the history of oxygen‐sensitive enzymes such as Rubisco.

## 5 CONCLUSIONS

We reconstructed the ancient Rubisco variants representing five different time points that traverse the rise of significant levels of oxygen over Earth's past. By comparing sequences along internal branches of the family phylogeny, we revealed a map of amino acid substitutions connecting diverse Rubisco genotypes. Analysis of the rate of historic substitution rates with respect to their structural locations shows that the Rubisco family experienced distinct mutational enrichments at its active site, subunit interface, and various dimer interfaces just prior to the emergence of recognizable Form I ancestral sequences; there were no such periods of markedly increased substitution on rates before or after this period.

The reconstruction of ancient Rubisco protein mutational trajectories yields a number of testable hypotheses. The site substitutions inferred for the N‐terminus region between the Anc. I/III and Anc. I/III’ node sequences should be tied to variable expression of Rubisco that optimizes carboxylation in anaerobes under oxidative stress. Functional variants of sequences closely related to anoxygenic archaeal and bacterial ancestral precursors should exhibit functional optimality under high CO_2_/low O_2_ partial pressure conditions. Finally, decreased CO_2_/O_2_ specificity for anoxic ancestral Rubisco sequences should coincide with decreased carbon isotope fractionation associated with carboxylation activity (Boller, Thomas, Cavanaugh, & Scott, 2015), which may have profound implications for the interpretation of organic carbon isotope ratios on the early Earth (Bell, Boehnke, Harrison, & Mao, 2015; Schidlowski, 2001; Schopf, 2001). Testing these hypotheses may shed light on the delicate balance between Precambrian organismal metabolism and global‐scale geochemical fluxes. Regardless of the mechanisms invoked, it is clear that mapping expressly cyanobacteria‐derived bioinformatic and biochemical data onto biogeochemical events surrounding the GOE should be undertaken with great care in light of poor chronological constraints on phylogenetic and paleoenvironmental uncertainties. For these reasons, attempts to map phenotypic attributes of recently derived Rubisco clades to first‐order geochemical or macroevolutionary events are likely to be compromised without considering the full range of adaptations involved with accommodating Rubisco's transition from anoxia to oxia.
