# Supplementary material for: Constraining the timing of the Great Oxidation Event within the Rubisco phylogenetic tree
Source: Geobiology. 2017 Jul 3;15(5):628–40. doi: 10.1111/gbi.12243 (PMC5575542; doi:10.1111/gbi.12243)
Supplement: Supplementary file 1 [file GBI-15-628-s001.PDF]

SUPPLEMENTAL INFORMATION

Sequence Curation

The eighty-one protein sequences used in this study are available to download from the following URL: http://www.phylobot.com/582058404/RuBisCO.noalign.fasta

Maximum Likelihood Phylogeny

The maximum likelihood phylogeny used in this study is listed below. Decimals on internal branches express statistical support as approximate likelihood ratios (aLRs).

((B.Selaginella.Plant.Fern:0.02734201,(B.Nicotiana.Plant.tabacco:0.05407292,B.Spinaci
a.Plant:0.04776205)'5.9363e+06':0.02809071)'1.1043e+03':0.01241794,(B.Euglena.prot
ist:0.07372306,(((B.Trichodesmium.Cyanob:0.07092371,(B.Gloeobacter.Cyanob:0.0887
6145,(((B.Synechococcus.Cyanob:0.08536662,(((CD.Burkholderia.BetaP:0.10838969,(((
(CD.Nitrosospira.BetaP:0.04627936,CD.Nitrosococcus.GammaP:0.05090836)'6.5207e+
02':0.03840129,(CD.Porphyridium.alga:0.05549465,((CD.Pleurochrysis.alga:0.03147954
,CD.Emiliania.Diatom.Phytoplankton.Eu:0.04401492)'1.8156e+29':0.07232853,(((CD.Cyl
indrotheca.alga:0.03748225,CD.Odontella.alga:0.04311378)'6.6945e+24':0.06755264,C
D.Olisthodiscus.alga:0.07533440)'5.6746e+01':0.01939917,CD.Ectocarpus.alga.brown:0
.11649879)'1.2086e+12':0.03340696)'9.2074e+00':0.01350633)'3.1451e+31':0.1261375
3)'1.7911e+10':0.09022158,((CD.Nitrobacter.AlphaP:0.07710559,CD.AII.Rhodopse.Alph
aP:0.06299214)'1.7547e+06':0.04279121,CD.Acidiphilium.AlphaP:0.10062566)'3.9859e
+05':0.04997561)'2.4579e+14':0.09523292,(CD.Rubrivivax.BetaP:0.14622171,(CD.Rho
dobacter.AlphaP:0.05745568,(CD.Paracoccus.AlphaP:0.09454899,CD.Sinorhizobium.Al
phaP:0.04276247)'2.9766e+12':0.05630703)'3.4811e+13':0.06667397)'1.6041e+12':0.0
6910602)'3.2827e+02':0.05314065)'2.2897e+12':0.27138800,((III2.Hyperthermus.Archa.
Cren:0.23860548,((III2.Archaeoglobus.Archa.Eu:0.20579080,III2.Thermococcus.Archa.
Eu:0.24792436)'3.6956e+09':0.14714007,III2.Thermofilum.Archa.Cren:0.27697427)'9.64
96e+13':0.23234641)'7.9348e+06':0.23926857,(((((IV.Bordetella.bronchiseptica:0.59374
573,(IV.Octadecabacter.antarcticus:0.54120206,IV.Variovorax.paradoxus:0.40274523)'1
.2119e+04':0.12128963)'1.5535e+01':0.22992453,IV.Bacillus:0.42766617)'4.3696e+25':
0.62356580,(((IV.Chlorobium.tepidum:0.03238214,IV.Chlorobaculum.thiosulfatiphilum:0.
03051955)'4.0104e+07':0.21704983,(IV.Allochromatium.vinosum:0.31915431,IV.Rhodo
pseudomonas.palustris:0.25201081)'5.0252e+00':0.14384325)'4.4499e+49':1.09326034

,(IV.Tepidanaerobacter.acetatoxydans:0.77843306,IV.Archaeoglobus.fulgidus:0.556727
69)'2.3777e+11':0.43611300)'1.0000e+00':0.15500602)'7.9759e+10':0.37227348,(II.Mag
netospirillum.AlphaP:0.13468620,(II.Rhodospirillum.AlphaP:0.16541801,(IV.Symbiodiniu
m:0.97093144,(((II.A.Hydrogenovibr.GammaP:0.35663384,(II.Thiomicrospira.GammaP:
0.16102538,(II.A.Thiobacillus.BetaP:0.04912429,(II.Rhodoferax.BetaP:0.13026428,(II.P
olaromonas.BetaP:0.10982543,II.Dechloromonas.BetaP:0.10158927)'2.3933e+10':0.06
865894)'1.4746e+00':0.02765263)'2.0287e+06':0.07656150)'5.5097e+02':0.07191213)'1
.6483e+24':0.14963122,II.ACD.Rhodopse.AlphaP:0.13850116)'3.3988e+05':0.03768070
,II.A.Rhodobacter.AlphaP:0.10603257)'1.1153e+01':0.01994737)'3.3121e+01':0.070630
61)'3.2068e+00':0.09830042)'2.4827e+69':1.45218692)'3.4480e+04':0.24940636,((III1.
Methanosarcina.Archa:0.66042813,III1.Methanocaldococcus.Archa:0.52969026)'1.6000
e+02':0.13448452,((III2.Methanoculleus.Archa.Eu:0.54226271,III2.Pyrococcus.Archa.Eu
:0.37922705)'1.0663e+03':0.15155301,III2.Natronomonas.Archa.Eu:0.82872468)'2.0799
e+00':0.07418523)'3.4349e+03':0.14223958)'1.3826e+01':0.10275554)'1.2980e+81':0.9
0263288)'2.2663e+12':0.27453366,((((A.Acidithiobacillus.GammaP:0.08532258,(A.Nitro
bacter.AlphaP:0.09830112,(((A.Alkalilimnicola.GammaP:0.08429208,(A1.Thiomicrospira
.GammaP.Hydrothermal.SulfurOx:0.09654806,((A.CDII.Rhodopse.AlphaP.purpleNonsulf
ur:0.13547054,A.Hydrogenophilus.BetaP.Thermophilic:0.08546874)'1.1820e+02':0.0317
2817,A.II.Rhodobacter.AlphaP:0.08134808)'1.0000e+28':0.08211211)'2.4014e+02':0.02
420530)'7.2962e+07':0.04140940,(A.Methylococcus.GammaP.Methanotrophic:0.041831
88,A.Halorhodospira.GammaP:0.09100754)'2.7655e+02':0.02177177)'1.1471e+04':0.03
336367,(A1.Allochromatium.GammaP:0.06584666,(A.II.Thiobacillus.BetaP:0.02585648,(
A.Nitrosomonas.BetaP:0.07987768,A.Cupriavidus.BetaP:0.05119813)'4.5915e+01':0.01
273176)'8.0177e+00':0.02227756)'5.3353e+05':0.02844446)'5.7017e+00':0.01769782)'9
.6546e+03':0.03178906)'1.9390e+16':0.10142700,(A.Synechococcus.Cyanob:0.025470
97,A.Prochlorococcus.Cyanob:0.04409762)'2.5661e+35':0.13458502)'1.4378e+01':0.04
207085,A2.Allochromatium.GammaP:0.11013450)'2.0854e+03':0.03803794,(A2.Thiomic
rospira.GammaP.Hydrothermal.SulfurOx:0.02244319,A.II.Hydrogenovibr.GammaP.Hydr
ogenOxidizing:0.01181450)'6.0321e+15':0.06959093)'8.9458e+10':0.12119554)'7.3018e
+02':0.04517083)'9.4018e+06':0.04285749,B.Thermosynechococ.Cyanob:0.07096793)'
3.2234e+02':0.02302577,(B.Nostoc.Cyanob:0.01607461,B.Anabaena.Cyanob:0.033179
05)'3.5924e+10':0.04319178)'1.5627e+01':0.01827782)'5.9387e+06':0.03137530)'3.524
5e+00':0.01283384,(B.Prochlorothrix.Cyano:0.05996676,B.Synechocystis.Cyanob:0.071
41728)'1.7334e+02':0.02236275)'8.5327e+42':0.10824674,(B.Chlamydomonas.Alga:0.0

3109527,(B.Ostreococcus.Alga:0.08413521,B.Chlorella.alga:0.03826385)'8.2238e+01':0.01049030)'5.1906e+04':0.01046158)'3.3510e+02':0.01010081)'1.3526e+17':0.04325088,B.Physcomitrella.Plant.Moss:0.03924231);

Maximum Likelihood Ancestral Protein Sequences

Anc. I/II/III:
MEVHDRYRKLDFVDPDYVPGDYLICTYRIEPAEGISIEEAAARIASESSTGTWTTVSTTEELRERLKAKVYDIEEIGDHSYLVKIAYPLELFEEGSLPNLLSTIAGNIFGMKAVKGLKLLDLHFPPAFLKTFKGPKFGIEGVREILGVKDRPLLGTVIKPKVGLSPEEYAKVAYEVWLGGIDFIKDDELLANQPFCRFEERVKKVMEAIDRAEEETGERKLYLVNITAPVDEMVKRAELVVDYGGNCVMIDIVTAGWSALQTLREHeDLDLAIHAHRAGHGAFTRNPKHGISMLVLAKLARLAGVDHIHTGTAGYGKMEGNEEEVLEIAKMLREDEADGFFLEQDWGNIKPCFPVASGGLHPGTVPELIDTLGKDIIIAAGGGIHGHPDGPAAGARAMRQAIEAVMEGVSLDEYAKEHPELKRALEKWGHAAR

Anc. I//III:
MHEHDRYLDFVDPNYKPGDDLICTFRIEPAEGISIEEAAARIASESSTGTWTTLSTKPEMMERLKAKVYDIEEIGDGSYLVKIAYPLELFEEGSIPNLLSSIAGNIFGMKAVKGLRLLDLHFPAAYLKTFKGPQFGIEGVREILGVKDRPLLGTVPKPKVGLSPEEYAKVAYEVWMGGIDFVKDDENLASQPFCRFEERVKKVMKAIDRAEEETGERKAYLVNITAPVEEMVKRAELVADYGGNYVMIDIVTAGWSALQTLRELAEDLDLAIHAHRAMHAAFTRNPKHGISMLVLAKLARLAGVDHIHTGTAGVGKMEGNKEEVLEITDILREEHYDEGDGFFLEQDWHNIKPVFPVASGGLHPGTVPELIDILGKDIIIQAGGGVHGHPDGPRAGARAMRQAIEAAMEGVSLDEYAKEHPELKRALEKWGHVKR

Anc. I/III’ (the immediate descendant from Anc. I/III):
MHEHFDSYLEFVDPNYKPGDDLIAVFRITPAEGISIEEAAARIAAESSTGTWTTLSTEKPSMMERLKAKAYDIEELGDGSYLVRIAYPLELFEEGSIPNLLSSIAGNIFGMKAVKGLRLEDLHFPASYLKTFKGPQFGIEGVREILGIKDRPLLGTVPKPKVGLSPEEYGKVAYEVLIGGIDFVKDDENLASQPFCRFEERVKKVMKAIDRAEEETGERKAYLVNITAPVEEMVKRAELVADYGGNYVMIDIVTAGWSALQTLRELAEDYDLAIHAHRAMHAAFTRNPKHGISMLVLAKLARLAGVDHIHTGTAgVGKMEGKKREVLEITDILREQHYKPDEGDGFFLEQDWSNIKPVFPVASGGLHPGTLPELIDILGKDIIIQVGGGVLGHPDGPRAGARAVRQAIEAAMEGISLDEYAKEHPELKRALEKWGHVKV

Anc. I:

MNETRYKAGVKKYRLGYWEPDYTPKTDLIAAFRITPQPGVPPEEAAAAVAAESSTGTW
TTVWTDRLTALDRYKAKAYRIEPVPGEEGSYFAYIAYPLDLFEEGSVANLLTSIVGNVFG
FKALKALRLEDIRFPVAYVKTFQGPPTGIQVERERLDKYGRPLLGATVKPKLGLSAKNY
GRVVYECLRGGLDFMKDDENINSQPFMRWRDRFLFVMEAVNKAQAETGEVKGHYLN
VTAPTMEEMYKRAEFAKELGSPIIMIDLVTAGWTAIQTLAKWARDNDMLLHLHRAMHAT
YTRQKNHGISFRVLAKWLRLAGVDHLHTGTAVGKLEGDPASVLGFVDVLRESYIEVDR
SRGIFFDQDWASLRKVMPVASGGIHAGQMPQLVDIFGDDVVLQFGGGTLGHPAGNQA
GATANRVALEAMVKARNEGRDLVREGPDILREAAKWSPELKQALETWKDVKSDPDVV
ETATAA

Anc. IA/B:

MSKTTYSKAGYQAGVKDYRLTYWTPDYTPKTDLLAAFRVTPQPGVPPEEAAAAVAAES
STGTWTTVWTDLLTDLDRYKGRCYRIEPVPGEENSYFAFIAYPLDLFEEGSVTNVLTSIV
GNVFGFKALRALRLEDIRFPVAYVKTFQGPPHGIQVERDKLNKYGRPLLGCTIKPKLGLS
AKNYGRAVYECLRGGLDFTKDDENINSQPFQRWRDRFLFVAEAIHKAQAETGEIKGHY
LNVTAPTCEEMMKRAEFAKELGMPIIMHDFLTAGFTANTTLAKWCRDNGMLLHIHRAM
HAVIDRQKNHGIHFRVLAKCLRLSGGDHLHTGTVVGKLEGDRASTLGFVDLLRESYIEE
DRSRGIFFDQDWASMPGVMAVASGGIHVWHMPALVEIFGDDSVLQFGGGTLGHPWG
NAAGATANRVALEACVQARNEGRDLMREGGDILREAAKWSPELAAALETWKEIKFEFD
VVEAMDTL

Anc. IB:

MSKTYQSKAGYQAGVKDYRLTYYTPDYTPKTDLLAAFRVTPQPGVPPEEAGAAVAAES
STGTWTTVWTDLLTDLDRYKGRCYHIEPVPGEENSYFAFIAYPLDLFEEGSVTNVLTSIV
GNVFGFKALRALRLEDIRFPVAYVKTFQGPPHGIQVERDKLNKYGRPLLGCTIKPKLGLS
AKNYGRAVYECLRGGLDFTKDDENINSQPFQRWRDRFLFVADAIHKAQAETGEIKGHY
LNVTAPTCEEMMKRAEFAKELGMPIIMHDFLTAGFTANTTLAKWCRDNGMLLHIHRAM
HAVIDRQKNHGIHFRVLAKCLRLSGGDHLHTGTVVGKLEGDRASTLGFVDLLRENYIEQ
DRSRGIFFTQDWASMPGVMAVASGGIHVWHMPALVEIFGDDSVLQFGGGTLGHPWG
NAPGATANRVALEACVQARNEGRDLMREGGDILREAAKWSPELAAALELWKEIKFEFE
AMDTL

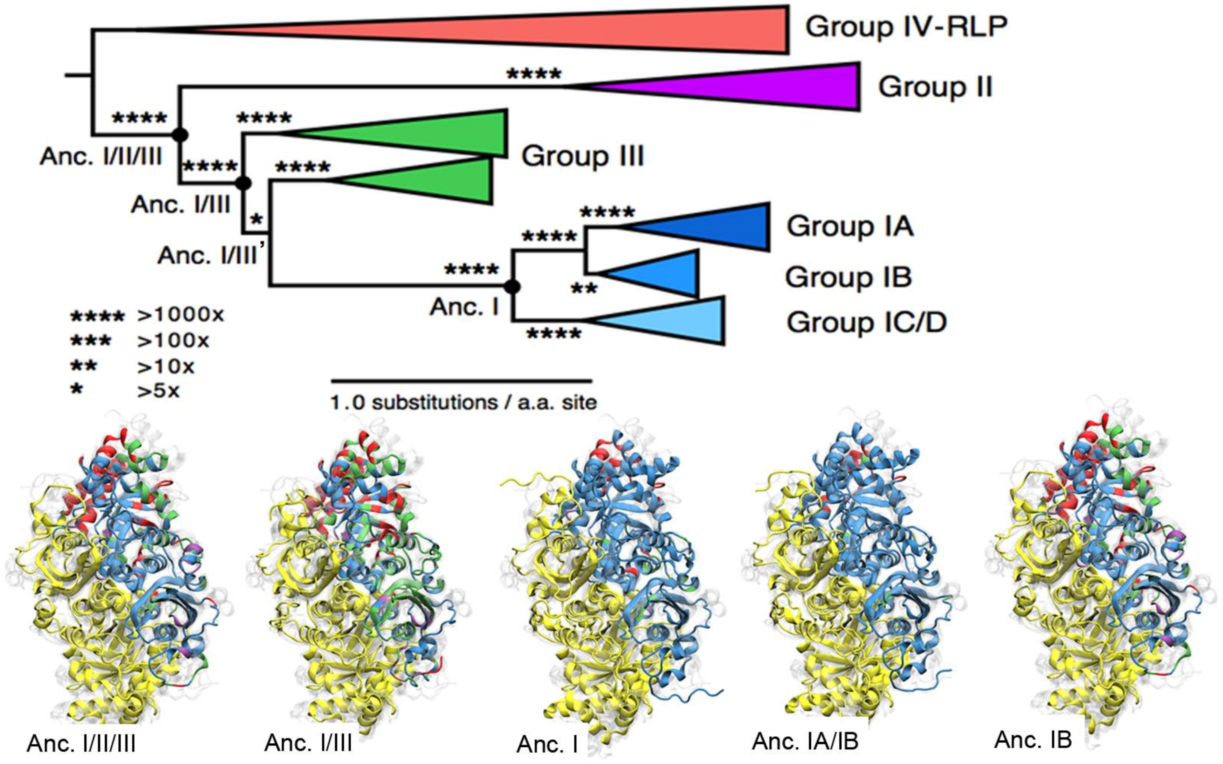

Figure SI-1. Top: A color-coded Rubisco phylogeny. Bottom: Ancestral Rubisco large subunits color-coded by closest distance of individual ancestral amino acid sites to corresponding residues from Form 1 (blue), Form II (purple), Form III (green) or Form IV (red) representative structures.

Alignment Method	Evolutionary Model	log(Likelihood)	Relative Probability	Alpha	Sum of Branch Lengths
msaprobs	PROTGAMMALG	-33409.65	0.2	1.056	28.49
msaprobs	PROTGAMMAWAG	-33598.16	0.1	1.189	24.17
msaprobs	PROTCATLG	-32867.51	0.3	1	28.52
msaprobs	PROTCATWAG	-33057.01	0.3	1	24.03
msaprobs	PROTGAMMAJTT	-34014.25	0	1.023	27.59
msaprobs	PROTCATJTT	-33484.48	0.1	1	27.40
muscle	PROTGAMMALG	-33228.64	0.2	1.071	28.72
muscle	PROTGAMMAWAG	-33423.97	0.1	1.192	24.27
muscle	PROTCATLG	-32701.11	0.3	1	28.46
muscle	PROTCATWAG	-32898.35	0.2	1	24.26
muscle	PROTGAMMAJTT	-33847.07	0	1.018	28.60
muscle	PROTCATJTT	-33043.97	0.2	1	28.28

Table SI-1. Comparison of models and methods used to reconstruct the Rubisco family evolution. We tested all combinations of two alignment methods (MSAProbs[Liu et al., Bioinformatics 2010], Muscle[Edgar, Nuc. Acids Research 2004]) with six evolutionary models implemented in RAxML (Stamatakis, Bioinformatics 2006). The method-model combination used in this paper (msaprobs + PROTCATWAG) is highlighted in yellow.

Ancestor	Mean Probability	Standard Deviation	URL
Anc. I/II/III	0.75	0.25	http://www.phylobot.com/rubisco.v4/msaprobs.PROTCATWAG/90.support
Anc. I/III	0.83	0.23	http://www.phylobot.com/rubisco.v4/msaprobs.PROTCATWAG/101.support
Anc. I	0.84	0.22	http://www.phylobot.com/rubisco.v4/msaprobs.PROTCATWAG/107.support
Anc. IA/B	0.95	0.12	http://www.phylobot.com/rubisco.v4/msaprobs.PROTCATWAG/124.support
Anc. IA	0.98	0.09	http://www.phylobot.com/rubisco.v4/msaprobs.PROTCATWAG/140.support
Anc. IB	0.98	0.09	http://www.phylobot.com/rubisco.v4/msaprobs.PROTCATWAG/125.support

Table SI-2. Summary of probability support for ancestral amino acid sequences. Every row corresponds to one ancestor in our study. Mean Probability is the average of posterior probabilities for the best amino acid at every site in the ancestral protein sequence. The link in the URL column points to an interactive visualization of the full probability distribution.

	Anc. I/II/III	Anc.I/ III	Anc. I	Anc. IA/B	Anc. IB	1GE H	1RBL	1TE L	9RU B
**Anc. I/II/III**	0.00								
**Anc. I/III**	0.10	0.00							
**Anc. I**	0.47	0.44	0.00						
**Anc. IA/B**	0.23	0.16	0.38	0.00					
**Anc. IB**	0.23	0.17	0.38	0.00	0.00				
**1GEH**	0.96	1.04	1.16	1.17	1.17	0.00			
**1RBL**	0.55	0.51	0.45	0.35	0.35	1.35	0.00		
**1TEL**	1.00	1.07	1.36	1.13	1.13	1.27	1.57	0.00	
**9RUBfig**	1.43	1.46	1.39	1.24	1.24	2.31	1.32	2.39	0.00

Table SI-3. Average Distances (Angstroms) between Rubisco structures. Distance is expressed as the root-mean-square deviation (RMSD) of atomic positions, measured in Angstroms. Every cell expresses the RMSD between two structures. Structures are named in rows and column headers according to their ancestral name, or their extant name.

Fisher Exact P-Values:	Form I L-L Interface	Form I L-S Interface	Form III L-L Interface	Dimer Interface	near AB barrel	in AB barrel	loop 6	In N-term domain	In C-term domain
Anc. I/II/III to Anc. I/III	0.310	0.244	0.386	0.095	0.171	0.152	0.349	0.144	0.144
Anc. I/III to Anc. I/III'	0.528	0.206	0.350	0.029	0.226	0.381	0.655	0.011	0.011
Anc. I/III' to Anc. I	0.003	0.003	0.122	0.015	0.013	0.010	0.064	0.026	0.026
Anc. I to Anc. IAB	0.225	0.125	0.246	0.017	0.109	0.197	0.340	0.067	0.067
Anc. IAB to Anc. IB	0.594	0.257	0.671	0.201	0.060	0.492	0.805	0.269	0.269

Odds Ratios:	Form I L-L Interface	Form I L-S Interface	Form III L-L Interface	Dimer Interface	near AB barrel	in AB barrel	loop 6	In N-term domain	In C-term domain
Anc. I/II/III to Anc. I/III	0.000	0.911	1.045	1.698	1.209	2.016	1.740	1.363	0.734

Anc. I/III to Anc. I/III'	0.000	1.578	1.710	0.000	0.880	0.846	0.000	3.671	0.272
Anc. I/III' to Anc. I	3.403	2.079	1.539	0.632	1.538	2.290	0.200	0.702	1.425
Anc. I to Anc. IAB	0.781	1.204	0.663	0.508	0.865	1.011	0.600	0.721	1.388
Anc. IAB to Anc. IB	0.000	1.693	0.000	0.339	0.000	0.000	0.000	1.180	0.847

Table SI-4. Thermal map of ancestral branches and protein structural elements that underwent mutational enrichment relative to background (thick bordered boxes), as indicated by our selected cut-offs for Fisher Exact P-values (top) and Odds Ratios (bottom).
